# A survey assessing the impact of symptoms related to the menstrual cycle and perceptions of workplace productivity: considerations for employer-sponsored menstrual health programs

**DOI:** 10.1186/s12905-025-03833-w

**Published:** 2025-08-30

**Authors:** Danielle M. Raves, Wynetta D. Herrera, Matthew E. Darnell, Tristan Rice, Craig Friedman, Stephanie C. Moratti, Stacy T. Sims, Wandasun B. Sihanath, Shannon N. Ehrhardt, Amanda Phillips

**Affiliations:** 1 Exos, 2629 E Rose Garden Lane, Phoenix, Arizona 85050 USA; 2 AUT Sports Performance Research Institute, Auckland University of Technology, Auckland, New Zealand; 3 Stanford Lifestyle Medicine, Stanford University, Stanford, CA USA; 4 Marquette University, Milwaukee, WI USA; 5 Sonoran University of Health Sciences, Tempe, AZ USA

**Keywords:** Work-related productivity, Menstrual health programming, Menstrual-related symptoms, Hormone-related symptoms, Menstrual cycle, Female health

## Abstract

**Background:**

Hormonal-related symptoms experienced during natural or contraceptive-driven menstrual cycles have implications on work-related productivity; however, employer-sponsored menstrual health resources are widely unavailable. Actionable research-based evidence is needed to develop menstrual health programs that proactively help working females mitigate their hormonal-related symptoms and optimize their hormone profiles and work-related performance. This study sought to evaluate the prevalence and severity of hormonal-related symptoms and assess the directional impact of hormonal-related symptoms on work-related productivity across cyclical hormone phases.

**Methods:**

A cross-sectional, descriptive questionnaire was used to measure hormonal-related symptoms and work-related productivity in 372 working females of reproductive age in the United States. The validated Menstrual Distress Questionnaire was used to measure the prevalence and severity of hormonal-related symptoms. The Menstrual Cycle-Related Work Productivity Questionnaire was modified and used to assess perceptions of work-related productivity measures across all cyclical hormone phases. Cumulative link mixed models and Bayesian adjacent category models were employed to determine the relationship between hormonal-related symptoms and work-related productivity, independent of age, body mass index (BMI), heavy bleeding experience, cyclical hormone phase, contraceptive use, Exos employment status and other hormonal-related symptoms.

**Results:**

Hormonal-related symptoms were present across cyclical hormone phases, and the most severe disturbances were experienced during the bleed-phase. Distributions of perceived work productivity were significantly more negative during the pre-bleed and bleed phases and more positive during the late follicular and early luteal phases. Self-reported hormonal-related symptoms were significantly associated with perceptions of work-related productivity, independent of potential confounders.

**Conclusions:**

Cyclical hormone fluctuations impact perceived work-related productivity variably by phase. Self-reported hormonal-related symptoms are associated with perceptions of work-related productivity. Our findings identify important considerations for the development of menstrual health programming to optimize the lived experience of female physiology in the workplace.

**Supplementary Information:**

The online version contains supplementary material available at 10.1186/s12905-025-03833-w.

## Background

Nearly 60 million females of reproductive age work in the United States (U.S.) [1]. The presence and severity of menstrual-related symptoms and hormonal-related symptoms, which are symptoms caused by hormonal fluctuations occurring in natural and contraceptive-driven menstrual cycles, are expansive, diverse, and unique among females [2]. Up to 91% of women studied worldwide have reported experiencing menstrual pain, and 29% report severe pain [3, 4]. Menstrual-related symptoms not only impact females physically, emotionally, and psychologically, but have negative implications on many functional outcomes, including quality of life, work productivity, and social relationships [5–15]. Menstrual-related symptoms can have significant economic consequences for women, employers, and healthcare systems [9]. In the U.S., general productivity loss costs employers $225.8 billion dollars annually [15] and older estimates put the annual indirect costs associated with menstrual-related symptoms at $4,333 per person [16]. Despite the costs, employer-sponsored menstrual health benefits are severely limited [13]. Developing, validating, and implementing menstrual health programming is essential to address a fundamental health issue and to help close a widely acknowledged gap in, not only the literature, but in the current real-life everyday experiences of the nearly 60 million working females of reproductive age in the U.S.

In a study of 1,867 working U.S. women, 45.2% reported missing work (5.8 average days) in the past year due to their menstrual cycle [13]. While absenteeism affects work productivity, presenteeism is a larger contributor to work-related productivity loss [14, 15, 17]. In a cohort of 32,748 Dutch women, 80.7% reported presenteeism due to menstrual-related symptoms with those experiencing abdominal pain losing 1.3 days of productivity to absenteeism versus 8.9 days to presenteeism [18]. However, within presenteeism, it is unclear which productivity measures are drivers, and which menstrual-related symptoms or hormonal-related symptoms coincide with changes in productivity. Associations between presenteeism and menstrual-related symptoms are limited primarily to within-week evaluations, mainly the pre-menstrual or menstrual phases. Research has confirmed the impact of menstrual-related symptoms on work-related productivity [5–15], but actionable evidence is needed to develop programs that help working females optimize their experience and mitigate symptoms [19].

Ponzo et al. [13] evaluated the negative impact of the menstrual cycle on work productivity in a group of working U.S. women. Most participants reported a moderate-to-severe negative impact across work productivity measures [13]. To our knowledge, this was the first study to evaluate the impact of the menstrual cycle on multiple work-related productivity measures [13]. However, there was no differentiation between menstrual cycle phases or consideration of positive influences of the menstrual cycle on productivity [13]. A comprehensive understanding of menstrual-related symptoms or hormonal-related symptoms and the bi-directional impact on work-related productivity throughout cyclical hormone fluctuations is limited, as is assessing the impact and receptivity of employer-sponsored menstrual health programming in the U.S. In one study, 94.6% of participants reported that their employers did not provide benefits for menstrual-related issues; 75.6% who did not receive menstrual-related benefits reported wanting them [13]. Therefore, this study aimed to (1) evaluate the prevalence and severity of hormonal-related symptoms across all four phases of cyclical hormone fluctuations, (2) understand the phase-specific impact of these fluctuations on perceptions of work-related productivity, (3) measure hormonal-related symptom impact on perceptions of work-related productivity, and (4) identify the availability of employer-sponsored menstrual health programming and the receptivity to engage in these programs among working U.S. females of reproductive age.

## Methods

### Study design

The Exos Female Physiology Questionnaire Study was a cross-sectional, descriptive questionnaire-based study of females of reproductive age, including women, non-binary, and third-gender women who menstruate (those with a natural menstrual cycle and those who are contraceptive-driven), and perimenopausal females. The Exos Female Physiology Questionnaire (EFPQ), the survey developed for use in this study, was deployed over two study phases. Phase 1 included females currently employed at Exos (U.S. based), and Phase 2 extended to females nationwide, independent of Exos employment. Phase 2 initiated upon Phase 1 completion. The data were collected from April to October 2023. This study was reviewed and exempt by the Advarra institutional review board (IRB), and written informed consent was obtained from all participants.

### Participants

The present analysis includes data from 372 females aged 22–53 years. The exclusion criteria included: not self-reporting as a female at birth, being younger than 18 or older than 65 years old, not currently living in the U.S., not being fluent in English, not currently a full-time employee, having experienced menopause, being currently perimenopausal or pregnant, having had a hysterectomy, being unwilling to identify current menstrual status, not having had a menstrual cycle in the previous two months, never menstruating, currently breastfeeding and not experiencing a menstrual cycle, and the unwillingness to identify history of hysterectomy. Participants who reported being of reproductive age or were unsure if they were perimenopausal or menopausal, and met all other eligibility criteria, were included in the final sample. Participants included those who reported currently using contraception, including oral contraceptives, intrauterine devices (hormonal or non-hormonal), shots, or implants, as well as those who reported not using these contraceptives.

### Data collection

Participants completed the one-time, 20-minute self-report EFPQ survey. Eligibility was determined through initial survey questions. The consent and survey were developed, administered, and managed digitally using Qualtrics. Unique identifiers, assigned to each subject, were used to track unique survey completions.

### Questionnaire items

The EFPQ (See Additional File 1, Appendix A) is a robust survey that pulled or modified questions from other studies, previously validated surveys, or were designed for original use in this study. For clarity, focus, and brevity, only survey questions relevant to the present analysis are described in Additional File 1, Appendix A. References for each EFPQ question that was leveraged from another source, are cited with each relevant question. Data from EFPQ questions that were not evaluated in the present study are not summarized or cited herein. The EFPQ was piloted in a small group of Exos-affiliated females for qualitative feedback to reduce researcher bias. Given the personal nature of the survey, each question was optional or included a ‘prefer not to say’ response option. The surveys used in this study are described in detail below.

### Demographics/Sociodemographics

Age, location, Exos employment status, English fluency, legal adult status by state or territory, birth month and year, biological sex, gender identity, ethnicity, race, height and weight, education level, and employment information were collected and used for eligibility, descriptive, and analytical purposes.

### Menstrual-related information

Age of menarche, historical heavy bleeding experience, menstrual pain prevalence, menstrual symptom management, current menstrual life stage, hysterectomy status, current contraceptive use, time to last menstrual cycle, menstrual-related work loss, cycle tracking frequency, hormone-related symptom prevalence and severity, pregnancy status information, willingness to receive menstrual-related information, employer-based menstrual-related benefits status, and interest in menstrual-related content, were collected for eligibility, descriptive, and analytical purposes.

### Low Energy Availability in Females Questionnaire (LEAF-Q)

The LEAF-Q, a tool described elsewhere, was developed and validated for use in female endurance and dance athletes to identify risk of developing low energy availability [20]. It consists of 25 items that assess menstrual function, injury history, and gastrointestinal symptoms [20]. One question from the LEAF-Q was used in the present study for eligibility purposes.

### Menstrual Distress Questionnaire (MDQ)

The MDQ is a validated tool for measuring the presence and intensity of cyclical menstrual symptoms in females and has been described elsewhere [21]. It consists of 47 items rated on a five-point scale from ‘not at all’ to ‘disabling’, yielding eight subscale scores and a total distress score. Hormonal-related symptoms are recalled over three time frames: last menstrual flow (menstrual), week prior to the last flow (premenstrual), and the remainder of the most recent menstrual cycle (intermenstrual).

### Menstrual Cycle-Related Work Productivity Questionnaire

The Menstrual Cycle-Related Work Productivity Questionnaire, developed by the women's wellness Flo Health app ("Flo App") and described elsewhere [13] was modified for use in the present study. The Menstrual Cycle-Related Work Productivity Questionnaire measures employment-related information and assesses how negatively the menstrual cycle impacts six dimensions of work productivity, including concentration, efficiency, energy levels, relationship with coworkers, level of interest in their own work, and mood at work [13]. The degree of negativity was measured on a scale from 1(not at all) to 5 (extremely) [13]. In the current study, the response options were expanded to include bipolar responses, from ‘extremely negative’ to ‘extremely positive’, and the six dimensions of work productivity were evaluated across four hormonal phases. A visual of the menstrual cycle phase division diagram developed by Solli et al., and described elsewhere [22], of four hormonal phases [bleeding (Phase 1), early in the cycle (Phase 2), late in the cycle (Phase 3), and 4 − 1 days before bleeding (Phase 4)], was provided to participants as reference. Participants were also asked about days of work missed due to cyclical hormone fluctuations in the past six months.

### Menstrual-related terminology

**Fig. 1:**
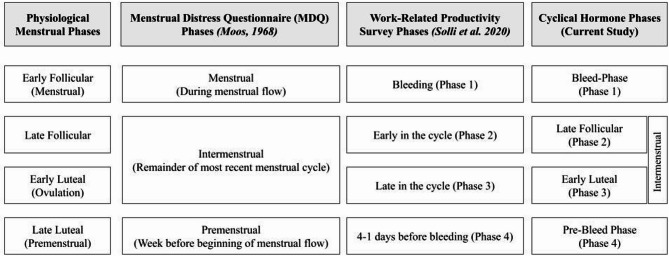
Menstrual phase description by source

The menstrual cycle consists of four phases: the early follicular phase (menstrual phase), the late follicular phase, the early luteal phase (ovulatory phase), and the late luteal phase (premenstrual phase) [23]. Since the current study included participants with natural and contraceptive-driven menstrual cycles, to more accurately account for participants with hormone-driven cycles the early follicular phase (menstrual phase) will be referred to herein as the bleed-phase or Phase 1 and the late luteal phase (premenstrual phase) will be referred to herein as the pre-bleed phase or Phase 4. Therefore, when pertaining to the outcomes from the MDQ, the three recall timeframes will be referred to herein as follows: bleed phase (last menstrual flow), pre-bleed phase (week prior to the last flow), and the intermenstrual phase (remainder of the most recent menstrual cycle). The four hormonal phases referenced in the modified Menstrual Cycle-Related Work Productivity Questionnaire will be referred to herein as follows: bleed phase (Phase 1), late follicular phase (Phase 2), early luteal phases (Phase 3), and pre-bleed phase (Phase 4). Figure 1 outlines the varying descriptions of menstrual or cyclical hormone phases by source, including: physiological menstrual phases, the MDQ menstrual phases developed by Moos [21], the menstrual phases by Solli et al. [22] used with the modified Menstrual Cycle-Related Work Productivity Questionnaire [13], and the hormone phase terminology described herein to account for participants with natural and contraceptive-driven cycles.

### Statistical analysis

Participants with complete demographic, MDQ, and work-related productivity data were used in this study. Body mass index (BMI) was imputed with the study population average when weight was not disclosed. Means and standard deviations were calculated for continuous variables and frequencies and percentages for categorical variables. MDQ symptom intensity was calculated as the average for participants who reported experiencing the symptom.

Average MDQ symptom intensity was compared across bleed-phase, pre-bleed, and intermenstrual phases using the Kruskal-Wallis test. To address multiple testing, false discovery rate (FDR) correction across the 47 Kruskal-Wallis tests was applied [24]. For significant hormone-related symptoms, pairwise comparisons between phases were performed using the Mann-Whitney U test with Bonferroni correction to adjust for multiple comparisons.

To assess the potential response bias from Exos employees (40.1%), a sensitivity analysis using the Kruskal-Wallis test was repeated in a sample limited to non-Exos employees and the results were compared to the overall sample. Results from the sensitivity analysis revealed systematic differences between non-Exos employees and the overall sample, prompting the inclusion of Exos employee status as a covariate in the subsequent ordinal logistic regression models to control for potential bias related to employment to the study organization.

The analysis of MDQ subscales was stratified by age and BMI category, and heavy bleeding experience. BMI categories were collapsed, combining underweight and healthy weight. For age and BMI, the Kruskal-Wallis test with FDR correction was used to assess overall differences across the three category groups, followed by pairwise comparisons via the Mann-Whitney U test with Bonferroni correction where appropriate. For heavy bleeding, which had only two categories, Mann-Whitney U tests were conducted for each outcome and corrected for multiple comparisons using FDR.

Differences in work-related productivity impacts across four hormonal phases were assessed. For each measure of work-related productivity, the Friedman test identified phase-related differences, with subsequent pairwise Wilcoxon Signed-Rank tests pinpointing specific contrasts within each outcome, adjusted via Bonferroni correction.

Ordinal logistic regression analyses, including cumulative link mixed models (CLMMs) and Bayesian adjacent category ordinal models, were used to examine the relationship between hormone-related symptoms, as assessed by the MDQ, and perceived impacts on six work-related productivity outcomes. Perceived impact was consolidated to a 3-point ordinal scale (‘negative’, ‘neither negative nor positive’, ‘positive’). All variables included in the models were pre-specified based on theoretical relevance and prior literature. Each model included fixed effects for age, BMI, cyclical hormone phase, current contraceptive use, heavy bleeding experience, Exos employment status, and relevant MDQ symptom intensity and subscale scores. MDQ scores were assigned by cyclical hormone phase: the bleed-phase (Phase 1), the intermenstrual phase (Phases 2 and 3), and pre-bleed phase (Phase 4). Phase 1 served as the reference level for all cyclical hormone phase comparisons. A random intercept at the subject level accounted for individual differences due to repeated measures. Continuous variables (e.g., age, BMI, symptom intensity, and MDQ subscales) were standardized prior to analysis. Categorical variables included contraceptive use, heavy bleeding experience, cyclical hormone phase, and Exos employment status.

Cumulative link mixed models (CLMMs) were used as the primary modeling approach. These models assume proportional odds, meaning the effect of each variable is consistent across thresholds of the ordinal outcome. For each model, Akaike Information Criterion (AIC) was used to evaluate model fit. Bootstrap resampling with 1,000 iterations was used to generate 95% confidence intervals (CIs), and results are presented as odds ratios (ORs) with corresponding CIs. To account for multiple comparisons across the six outcome models, Bonferroni correction was applied by setting the significance threshold at α = 0.0083 (i.e., 0.05/6) for the bootstrap-derived *p*-values.

To complement the primary CLMM results, a sensitivity analysis using Bayesian adjacent category ordinal models was conducted for each work-related productivity outcome. This approach evaluated the robustness of the CLMM findings and enabled more flexible estimation of non-proportional effects, specifically threshold-specific associations between adjacent outcome levels. These Bayesian models used an adjacent category logit link and incorporated partial proportional odds (POO) structures for variables where Brant tests and leave-one-out cross-validation (LOO) supported non-proportionality. Weakly informative priors (normal and Student-t) were used, and the resulting estimates were robust to prior variation. Convergence diagnostics confirmed reliability in each of our models. Statistical significance was determined by whether the 95% credible interval (CrI) for an odds ratio excluded one (1.0), indicating a high posterior probability of a non-null association. No formal correction for multiple comparisons was applied to these models, consistent with best practices in hierarchical Bayesian modeling, where partial pooling and regularization help mitigate the risk of false positives [25].

Based on effect sizes derived from prior literature, the sample size in this study is sufficient to achieve 80% power at a 0.05 significance level for the Mann-Whitney U and Wilcoxon signed-rank tests, as determined using G*Power. The assumptions for statistical tests were checked to ensure validity, and analyses were performed using R (version 4.4.0) and Python (3.9.18).

## Results

Descriptive statistics of the 372 participants are shown in Table 1. Additional education and work-related statistics for these participants are shown in Additional File 1, Appendix B. Every participant was employed full-time and 40.1% were Exos employees. The average age of the overall sample was 33.7 ± 7.0 years (range: 22–53 years) while the average age of non-Exos employees (*n* = 223) was 34.9 ± 6.3 (range: 22–53 years) and Exos employees (*n* = 149) was 32.0 ± 6.7 (range: 23–51 years). Nearly all (94.6%) reported being of reproductive age while 5.4% reported being unsure if they were perimenopausal or menopausal. The work environment distribution was onsite (51.6%), hybrid (24.2%), and remote (24.2%). Over half of participants (51.6%) reported ever having problems with heavy menstrual bleeding. Nearly all (89.5%) reported experiencing menstrual-related pain. Only 13.2% of all participants reported having missed work in the last six months due to their menstrual cycle, on average 2.8 days (range: 1–12 days). These findings were consistent in the non-Exos sample. Only 4.6% of participants reported that their employer offers any menstrual health benefits or wellness programs.

**Table 1:** Descriptive statistics of 372 full-time working females of reproductive age in the United States

	*n*	%	Mean ± SD
**Age**	372	-	33.7 ± 7.0
**BMI**	372	-	25.3 ± 4.9
**Gender Identity**			
Woman	371	99.7	
Non-Binary	1	0.3	
**Age Group**			
18 to 29	112	30.1	
30 to 39	189	50.8	
40 to 49	65	17.5	
50 +	6	1.6	
**BMI Category**			
Underweight	3	0.8	
Healthy Weight	211	56.7	
Overweight	108	29.0	
Obesity	49	13.2	
Prefer not to say	1	0.3	
**Race**			
American Indian or Alaskan Native	1	0.3	
Asian	31	8.3	
Black or African American	13	3.5	
Native Hawaiian or Other Pacific Islander	0	0.0	
White	295	79.3	
Other	14	3.8	
2 or more races	15	4.0	
Prefer not to say	3	0.8	
**Ethnicity**			
Hispanic or Latino	36	9.7	
Not Hispanic or Latino	331	89.0	
Prefer not to say	5	1.3	
**Work Environment**			
Onsite	192	51.6	
Hybrid	90	24.2	
Remote	90	24.2	

BMI, Body Mass Index; SD, Standard Deviation

The prevalence and intensity of hormonal-related symptoms experienced across hormone phases for the overall sample are shown in Table 2. Nearly all hormonal-related symptoms were most prevalent during the bleed phase, followed by the pre-bleed and intermenstrual phases, respectively. Each of the 47 MDQ symptoms were reported by participants during each hormone phase. At least 25% of participants reported experiencing ‘strong to disabling’ intensities of anxiety and changes in eating behaviors during their bleed-phase. The three most prevalent hormonal-related symptoms experienced during the bleed phase were fatigue (86.6%), cramps (uterine or pelvic) (86.3%), and change in eating habits (85.5%). By the intermenstrual phase, 46.8% and 48.9% of participants, respectively, reported experiencing fatigue and changes in eating habits, and 28.5% reported experiencing cramps.

**Table 2:** Menstrual Distress Questionnaire by cyclical hormone phase for the overall sample

Symptoms	Bleed-phase (*N* = 372)			Pre-bleed Phase (*N* = 372)			Intermenstrual (*N* = 372)			Significance Level			
	*n*	Prev	Int	*n*	Prev	Int	*N*	Prev	Int	Comparison Across Phases^2^	Bleed-phase vs. Pre-bleed Phase^3^	Bleed-phase vs. Intermenstrual^3^	Pre-bleed Phase vs. Intermenstrual^3^
Fatigue	322	86.6%	1.84	243	65.3%	1.54	174	46.8%	1.51	***	***	***	
Cramps (uterine or pelvic)	321	86.3%	1.96	164	44.1%	1.48	106	28.5%	1.53	***	***	***	
Change in eating habits	318	85.5%	1.94	251	67.5%	1.72	182	48.9%	1.45	***	**	***	***
Irritability	297^1^	80.1%	1.88	226	60.8%	1.76	133	35.8%	1.47	***		***	***
Mood swings	286	76.9%	1.77	228	61.3%	1.63	119	32.0%	1.45	**		***	
Anxiety	277	74.5%	1.99	240	64.5%	1.77	190	51.1%	1.47	***	**	***	***
General aches and pains	268	72.0%	1.59	169	45.4%	1.33	133	35.8%	1.23	***	***	***	
Affectionate	251	67.5%	1.75	201	54.0%	1.65	212	57.0%	1.58				
Backache	236	63.4%	1.82	146	39.2%	1.47	114	30.6%	1.47	***	***	**	
Headache	235	63.2%	1.77	148	39.8%	1.51	121	32.5%	1.36	***	**	***	
Decreased efficiency	229	61.6%	1.63	130	34.9%	1.38	77	20.7%	1.38	**	**	*	
Depression (feeling sad or blue)	227	61.0%	1.71	187	50.3%	1.55	107	28.8%	1.45	*		*	
Crying	222	59.7%	1.76	181	48.7%	1.63	94	25.2%	1.50	*		*	
Painful breasts/chest	214	57.5%	1.51	167	44.9%	1.47	62	16.7%	1.42				
Avoid social activities	213	57.3%	1.75	125	33.6%	1.65	97	26.1%	1.49	*		*	
Weight gain	211^1^	57.0%	1.45	146	39.2%	1.34	76	20.4%	1.37				
Difficulty concentrating	212	57.0%	1.66	138	37.1%	1.52	95	25.5%	1.36	*		**	
Feelings of well-being	194^1^	52.3%	1.59	150	40.3%	1.60	167	44.9%	1.91	***		***	**
Swelling (abdomen, breasts, or ankles)	193	51.9%	1.54	143	38.4%	1.39	67	18.0%	1.31	*		*	
Easily distracted	187	50.3%	1.60	129	34.7%	1.40	107	28.8%	1.35	*		*	
Insomnia (sleeplessness)	174	46.8%	1.59	122	32.8%	1.48	99	26.6%	1.44				
Muscle stiffness	173	46.5%	1.52	114	30.6%	1.29	101	27.2%	1.25	***	**	**	
Burst of energy or activity	171	46.0%	1.70	129	34.7%	1.57	169	45.4%	1.60				
Takes naps, stay in bed	170	45.7%	1.53	110	29.6%	1.42	81	21.8%	1.33				
Tension	163	43.8%	1.42	100	26.9%	1.35	84	22.6%	1.31				
Skin troubles - spots	162	43.5%	1.46	138	37.1%	1.51	101	27.2%	1.34				
Lowered school or work Performance	160	43.0%	1.53	90	24.2%	1.38	57	15.3%	1.32				
Forgetfulness	155	41.7%	1.46	99	26.6%	1.30	73	19.6%	1.32				
Restlessness	155	41.7%	1.54	107	28.8%	1.38	83	22.3%	1.30				
Loneliness	154	41.4%	1.74	116	31.2%	1.62	76	20.4%	1.43				
Excitement	118	31.7%	1.39	106^1^	28.6%	1.42	137	36.8%	1.66	**		**	*
Accident-prone (cut finger, break dish, etc.)	115^1^	31.0%	1.62	78	21.0%	1.49	54	14.5%	1.30				
Dizziness, faintness	95	25.5%	1.38	41	11.0%	1.39	33	8.9%	1.24				
Hot flashes	87	23.4%	1.56	44	11.8%	1.52	30	8.1%	1.37				
Confusion	84	22.6%	1.42	53	14.2%	1.38	38	10.2%	1.37				
Lowered motor coordination	84	22.6%	1.38	53	14.2%	1.34	37	9.9%	1.32				
Lowered judgment	77	20.7%	1.48	53	14.2%	1.45	33	8.9%	1.36				
Orderliness	76	20.4%	1.37	58^1^	15.6%	1.55	78	21.0%	1.58				
Blind spots, fuzzy vision	75	20.2%	1.48	35	9.4%	1.46	30	8.1%	1.23				
Nausea or vomiting	71	19.1%	1.38	37	9.9%	1.46	32	8.6%	1.25				
Heart pounding	66	17.7%	1.58	39	10.5%	1.38	29	7.8%	1.17	*		*	
Cold sweats	64	17.2%	1.61	36	9.7%	1.61	23	6.2%	1.39				
Feelings of suffocation	64	17.2%	1.69	39	10.5%	1.54	30	8.1%	1.40				
Chest pains	55	14.8%	1.47	32	8.6%	1.41	26	7.0%	1.27				
Staying at home from school or work	50	13.4%	1.60	24	6.5%	1.58	17	4.6%	1.29				
Numbness, tingling in hands or feet	49	13.2%	1.39	28	7.5%	1.29	23	6.2%	1.17				
Ringing in ears	47	12.6%	1.47	24	6.5%	1.29	24	6.5%	1.33				

Prev, Prevalence; Int, Average Intensity

^1^ *N* < 372

^2^ Significant difference by Kruskal-Wallis test with FDR correction

^3^ Significant difference by Mann-Whitney U pairwise comparison with Bonferroni correction

* *p*-value < 0.05; ** *p*-value < 0.01; *** *p*-value < 0.001

Comparisons across the three phases showed significant differences in the intensities of several affective and physical symptoms in the overall sample. Pairwise comparisons showed significant decreases in the average intensities reported in hormonal-related symptoms experienced between the bleed-phase and pre-bleed phase. Significant reductions (*p* ≤ 0.01) were observed in affective symptoms, including fatigue, anxiety, and decreased efficiency. Significant decreases (*p* ≤ 0.01) were observed in physical symptoms, including cramps, change in eating habits, general aches and pains, backache, headache, and muscle stiffness.

Pairwise comparisons showed hormone-related symptoms to have significantly lower average intensities during the intermenstrual phase compared to the bleed-phase. Significant decreases (*p* ≤ 0.05) in affective symptoms included: fatigue, irritability, mood swings, anxiety, decreased efficiency, depression (feeling sad or blue), crying, avoiding social activities, difficulty concentrating, and easily distracted. Significant decreases (*p* ≤ 0.05) in physical symptoms included: cramps, change in eating habits, general aches and pains, backache, headache, swelling (abdomen, breasts, or ankles), muscle stiffness, and heart pounding. The average intensity of excitement and feelings of well-being significantly increased (*p* ≤ 0.01) between the bleed-phase and intermenstrual phase. Pairwise comparisons showed significant decreases (*p* ≤ 0.001) in the average intensities of changes in eating habits, irritability, and anxiety experienced between the pre-bleed and intermenstrual phases. The average intensity of excitement and feelings of well-being significantly increased between the pre-bleed and intermenstrual phases (*p* ≤ 0.05 and *p* ≤ 0.01, respectively).

Results of the sensitivity analysis, shown in Additional File 1, Appendix C, revealed that only 8 of the 47 hormone-related symptoms remained significantly different across hormone phases in the non-Exos sample compared to 20 symptoms in the overall sample. The hormone-related symptoms that significantly differed across hormone phases in the non-Exos sample included fatigue, cramping, changes in eating habits, irritability, anxiety, general aches and pains, backache, and decreased efficiency. Differences in changes in eating habits, anxiety, and general aches and pains were no longer statistically different between the bleed and pre-bleed phases in the non-Exos sample. Similarly, the average intensities of backache and decreased efficiency were no longer significantly different between the bleed and intermenstrual phases.

The intensity of MDQ subscale scores across age and BMI categories, and heavy bleeding experience for the overall and non-Exos samples along with the details of these findings are found in Table 3. There was a significant inverse relationship (*p* ≤ 0.05) in many MDQ subscale intensities with age categories and a significant positive relationship with BMI categories and heavy bleeding experience in both samples. There were no significant differences in self-reported days of work missed as a result of the menstrual cycle across age, BMI, or heavy bleeding experience.

**Table 3:** MDQ subscale intensity scores across age and BMI categories and heavy bleeding in 372 working females for the overall and non-Exos samples

MDQ Subscale	Age Category						BMI Category						Heavy Bleeding			
	22–29		30–39		40+		Healthy		Overweight		Obese		Yes		No	
	*A*	*B*	*A*	*B*	*A*	*B*	*A*	*B*	*A*	*B*	*A*	*B*	*A*	*B*	*A*	*B*
Pain	8.87^1***,2**^	7.68^1***^	7.19^1**^	6.92	5.70	5.33	6.66^4**^	6.07	8.48	7.77	8.37	7.45	8.57^5***^	7.84^5***^	6.15	5.63
Concentration	6.23^1**^	6.34	5.03	4.63	4.20	4.10	4.92	4.56	5.43	5.27	6.22	5.61	6.35^5***^	6.05^5***^	4.01	3.72
Behavior Change	5.08^1***^	4.78^1***^	4.13^1*^	4.08^1**^	3.18	2.75	3.82^3*, 4*^	3.51	4.61	4.38	5.27	4.97	4.95^5***^	4.59^5***^	3.47	3.29
Autonomic	1.60	1.42	1.14	1.00	1.03	1.10	1.14	1.00	1.37	1.34	1.53	1.13	1.57^5**^	1.38^5*^	0.92	0.85
Negative	9.79^1***^	9.52^1***^	8.30^1*^	8.14^1*^	6.58	5.88	7.70	7.21	9.41	8.95	9.45	9.06	9.70^5***^	9.19^5**^	7.06	6.71
Water Retention	3.47	3.30	3.00	2.71	2.93	2.85	2.79^4**^	2.50^4**^	3.62	3.45	3.53	3.26	3.51^5**^	3.37^5**^	2.73	2.36
Arousal	4.54^1***,2***^	4.12	3.17	2.78	2.79	2.50	3.38	2.76	3.63	3.41	3.84	3.35	3.74	3.47^5*^	3.25	2.56
Control	2.24	2.06	1.68	1.43	1.25	1.29	1.70	1.55	1.87	1.61	1.88	1.42	2.20^5**^	1.82	1.30	1.25
MDQ Overall	41.82^1***,2*^	39.22^1***^	33.63^1*^	31.70	27.66	25.81	32.11^4*^	29.17	38.42	36.17	40.08	36.26	40.59^5***^	37.72^5***^	28.90	26.37

BMI, Body Mass Index; A, Overall Sample; B, Non-Exos Sample

¹Significant difference in age category vs. 40 + by Mann-Whitney U test with Bonferroni correction

²Significant difference in age category vs. 30–39 by Mann-Whitney U test with Bonferroni correction

^3^Significant difference in BMI category vs. obesity by Mann-Whitney U test with Bonferroni correction

^4^Significant difference in BMI category vs. overweight by Mann-Whitney U test with Bonferroni correction

^5^Significant difference in heavy bleeding vs. no heavy bleeding by Mann-Whitney U test with FDR correction

* *p*-value < 0.05; ** *p*-value < 0.01; *** *p*-value < 0.001

**Fig. 2:**
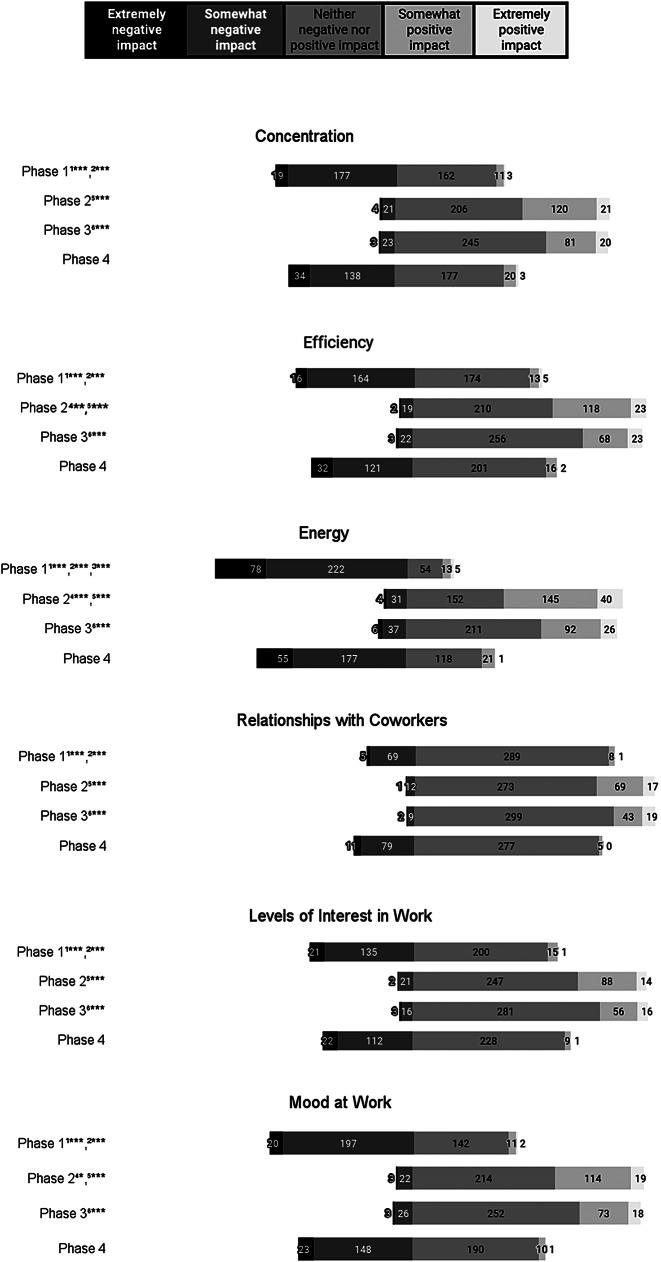
Perceived hormonal phase impact on areas of work-related productivity ^1^Significant difference in Phase 1 vs. Phase 2 by Wilcoxon Signed-Rank test with Bonferroni correction ^2^Significant difference in Phase 1 vs. Phase 3 by Wilcoxon Signed-Rank test with Bonferroni correction ^3^Significant difference in Phase 1 vs. Phase 4 by Wilcoxon Signed-Rank test with Bonferroni correction ^4^Significant difference in Phase 2 vs. Phase 3 by Wilcoxon Signed-Rank test with Bonferroni correction ^5^Significant difference in Phase 2 vs. Phase 4 by Wilcoxon Signed-Rank test with Bonferroni correction ^6^Significant difference in Phase 3 vs. Phase 4 by Wilcoxon Signed-Rank test with Bonferroni correction * *p*-value < 0.05; ** *p*-value < 0.01; *** *p*-value < 0.001

Results from the modified Menstrual Cycle-Related Work Productivity Questionnaire, including work-related productivity perceptions across the hormonal phases for the overall sample are shown in Fig. 2. Significant differences in response distributions were found across all six work-related productivity outcomes (*p* ≤ 0.001). Results showed significant changes in response distributions between Phases 1 and 2 and Phases 1 and 3 with greater positive perceptions of concentration, efficiency, energy, relationships with coworkers, mood at work, and levels of interest in work reported in Phases 2 (*p* ≤ 0.001) and 3 (*p* ≤ 0.001) compared to Phase 1.

Significant changes occurred in response distributions between Phases 2 and 4 and Phases 3 and 4 with greater negative perceptions of concentration, efficiency, energy, relationships with coworkers, mood at work, and levels of interest in work reported in Phase 4 and compared to Phases 2 and 3 (*p* ≤ 0.001). A significant change in response distributions existed between Phases 1 and 4 in perceptions of energy, with higher positive perceptions in Phase 4 compared to Phase 1 (*p* ≤ 0.001). There were significant changes in response distributions between Phases 2 and 3 with greater positive perceptions of efficiency (*p* ≤ 0.01), energy (*p* ≤ 0.001), and mood at work (*p* ≤ 0.05) in Phase 3 compared to Phase 2.

Frequentist ordinal logistic regression analyses (CLMMs) revealed significant associations between hormone-related symptoms and MDQ subscale scores with consolidated work-related productivity outcomes across cyclical hormone phases. These associations remained statistically significant after adjusting for multiple comparisons using Bonferroni correction across the six outcome models, and after controlling for covariates including age, BMI, current contraceptive use, heavy bleeding experience, cyclical hormone phase, and Exos employment status. ORs with 95% bootstrapped confidence intervals from the CLMMs are reported for all six models in Table 4.

**Table 4:** Model estimates from cumulative link mixed models for perceptions of work-related productivity outcomes

Variable	Concentration (*N* = 372)	Relationship with Coworkers (*N* = 372)	Energy (*N* = 372)	Efficiency (*N* = 372)	Mood at Work (*N* = 372)	Interest in Work (*N* = 372)
	OR, [95% CI]	OR, [95% CI]	OR, [95% CI]	OR, [95% CI]	OR, [95% CI]	OR, [95% CI]
Age	0.89, [0.75, 0.97]*	0.86, [0.69, 0.95]*	0.89, [0.75,0.98]*	0.87, [0.72, 0.93]*	0.98, [0.82, 1.09]	0.95, [0.79, 1.05]
BMI	1.11, [1.02, 1.31]*	1.06, [0.94, 1.26]	1.08, [0.96, 1.26]	1.06, [0.96, 1.25]	1.07, [0.96, 1.27]	1.11, [1.01, 1.35]*
Current contraceptive use	0.69, [0.47, 0.84]*	0.85, [0.56, 1.11]	0.93, [0.67, 1.21]	0.77, [0.55, 0.94]*	0.84, [0.59, 1.06]	0.75, [0.48, 0.88]*
Heavy bleeding experience	0.93, [0.73, 1.21]	0.95, [0.70, 1.33]	0.84, [0.61, 1.05]	0.95, [0.76, 1.24]	0.96, [0.76, 1.28]	0.87, [0.65, 1.14]
Exos employment status	1.16, [0.96, 1.65]	1.19, [0.93, 1.81]	0.97, [0.76, 1.34]	1.17, [0.95, 1.64]	1.02, [0.78, 1.42]	1.16, [0.90, 1.68]
Phase 2^a^	12.82, [14.45, 34.3]*	6.30, [5.89, 16.67]*	34.29, [47.94, 139.09]*	11.54, [12.42, 29.52]*	14.14, [16.75, 43.66]*	8.80, [8.87, 23.16]*
Phase 3^a^	8.60, [9.12, 20.67]*	4.47, [3.92, 10.97]*	18.37, [23.08, 63.5]*	6.71, [6.58, 14.97]*	9.03, [9.57, 23.79]*	6.41, [5.89, 14.73]*
Phase 4	1.09, [0.76, 1.64]	0.54, [0.26, 0.70]*	2.15, [1.68, 4.22]*	0.98, [0.64, 1.38]	1.32, [0.95, 2.06]	0.87, [0.51, 1.19]
Difficulty concentrating	0.68, [0.42, 0.79]*	-	-	0.88, [0.59, 1.13]	-	-
Avoid social activities	-	0.91, [0.60, 1.16]	-	-	-	-
Fatigue	-	-	0.73, [0.53, 0.78]*	-	-	-
Burst of energy or activity	-	-	1.40, [1.20, 2.12]*	-	-	-
Takes naps, stay in bed	-	-	0.80, [0.59, 0.91]*	-	-	-
Decreased efficiency	-	-	-	0.70, [0.47, 0.86]*	-	-
Mood swings	-	-	-	-	0.79, [0.52, 0.89]*	-
Staying at home from school or work	-	-	-	-	-	0.69, [0.42, 0.75]*
MDQ concentration subscale	0.89, [0.60, 1.15]	-	-	0.94, [0.60, 1.29]	-	0.85, [0.59, 1.04]
MDQ behavior change subscale	-	0.70, [0.44, 0.96]*	-	-	-	0.72, [0.43, 0.86]*
MDQ negative subscale	-	0.75, [0.50, 0.96]*	-	-	0.63, [0.39, 0.72]*	-
MDQ arousal subscale	-	1.38, [1.15, 1.75]*	1.00, [0.78, 1.31]	-	1.29, [1.18, 1.68]*	-

OR, Odds Ratio; CI, Confidence Interval

* Indicates statistical significance based on Bonferroni-adjusted bootstrap *p*-values (*p* < 0.0083), corrected across six outcome models

- Not included in model

^a^ Some Phase 2 and Phase 3 estimates showed evidence of inflation in bootstrap resampling and are addressed further using partial proportional odds Bayesian modeling (see Appendix D)

In the concentration model, Phase 2 and Phase 3 were significantly associated with a more positive impact on perceived work-related concentration as compared to Phase 1. Current contraceptive use, age, and higher intensities of self-reported difficulty concentrating were significantly associated with more negative perceptions of work-related concentration. BMI, MDQ concentration subscale score, Exos employment status, and heavy bleeding status were not statistically significant with perceptions of work-related concentration. There was no significant association between perceived work-related concentration in Phase 4 compared to Phase 1.

In the relationships with coworkers model, Phases 2 and 3 were significantly associated with more positive perceptions of relationships with coworkers, while Phase 4 was linked to a significant negative impact compared to Phase 1. Age was significantly associated with more negative perceptions of relationships with coworkers. Higher MDQ arousal scores were significantly associated with more positive perceptions of relationships with coworkers. BMI, current contraceptive use, heavy bleeding experience, Exos employment status, MDQ behavior change and negative subscale scores, and avoiding social activities were not statistically significant in the CLMM.

In the energy model, Phases 2, 3, and 4 were significantly associated with more positive perceptions of energy compared to Phase 1. Self-reported bursts of energy or activity were significantly associated with more positive perceptions, whereas fatigue and increased napping or staying in bed were significantly linked to more negative perceptions of energy. Age, MDQ arousal scores, BMI, current contraceptive use, heavy bleeding experience, and Exos employment status were not significant in the CLMM.

In the efficiency model, Phases 2 and 3 were associated with significantly more positive perceptions of work-related efficiency while Phase 4 was not significantly different, compared to Phase 1. Age and higher intensity of self-reported decreased efficiency were each significantly associated with more negative perceptions of work-related efficiency. Current contraceptive use, MDQ concentration subscale scores, difficulty concentrating, BMI, heavy bleeding experience, and Exos employment status were not significant with perceptions of work-related efficiency.

In the model for mood at work, Phases 2 and 3 were significantly associated with more positive perceptions of mood at work, while Phase 4 showed no significant difference, compared to Phase 1. Higher MDQ negative affect scores were significantly associated with more negative perceptions, while higher MDQ arousal scores were significantly associated with more positive perceptions of mood at work. Age, BMI, current contraceptive use, heavy bleeding experience, self-reported mood swings, and Exos employment status were not significant with perceptions of mood at work.

In the model for level of interest in work, Phases 2 and 3 were significantly associated with more positive perceptions of interest in work, while Phase 4 showed no significant difference, compared to Phase 1. Staying home from work or school due to symptoms and higher MDQ behavior change subscale scores were significantly associated with more negative perceptions of interest in work. Age, BMI, current contraceptive use, MDQ concentration subscale scores, heavy bleeding experience, and Exos employment status were not significant in the CLMM.

Bayesian partial proportional odds models produced findings that were highly consistent with the CLMMs. All effects that were statistically significant in the CLMMs were corroborated by the Bayesian models, with 95% credible intervals excluding one (1.0). Phases 2 and 3 remained significantly associated with more positive perceptions of work-related productivity across all outcomes, although OR estimates were generally lower than those from the frequentist CLMMs. Estimates for self-reported hormone-related symptom intensities, such as fatigue and burst of energy or activity were also directionally and statistically consistent between modeling approaches.

For several variables that were significant in both the frequentist and Bayesian models, the Bayesian partial proportional odds models revealed U-shaped, threshold-specific associations that were not detectable under the proportional odds assumption of the CLMMs. For example, in the efficiency model, higher intensities of self-reported decreased efficiency were associated with lower odds of neutral perceptions of efficiency compared to negative perceptions and higher odds of positive perceptions of efficiency compared to neutral perceptions. The MDQ negative subscale, composed of eight mood-related symptoms including irritability, depression, and anxiety, showed a significant U-shaped relationship with perceptions of mood at work. Participants with higher MDQ negative subscale scores were significantly less likely to report neutral perceptions of mood at work compared to negative perceptions and more likely to report positive perceptions of mood compared to neutral perceptions.

Similarly, the MDQ concentration subscale demonstrated a significant U-shaped association with perceptions of concentration such that higher MDQ subscale scores were associated with lower odds of reporting neutral perceptions of concentration compared to negative perceptions and higher odds of reporting positive perceptions of concentration compared to neutral perceptions. MDQ behavior change and negative subscales were significantly associated with coworker relationships at the lower threshold only, such that participants with higher MDQ subscales scores had higher odds of reporting negative perceptions as opposed to neutral perceptions of their relationships with coworkers. Age and MDQ arousal subscale were significantly associated with perceptions of energy, but at different thresholds. Age was significantly associated only at the higher threshold, such that older participants had higher odds of reporting more neutral perceptions of energy compared to positive perceptions. The MDQ arousal subscale showed a significant U-shaped relationship with perceived energy such that higher arousal subscale scores were associated with lower odds of reporting neutral perceptions of energy compared to negative perceptions and higher odds of reporting positive perceptions of energy compared to neutral perceptions.

Contraceptive use was significantly associated with lower odds of reporting higher levels of efficiency, indicating a consistent tendency toward more negative perceptions across outcome thresholds. Age and mood swings were both significantly associated with perceptions of mood at work, though their patterns differed. Age was significant only at the higher threshold with older females showing higher odds of reporting neutral perceptions of mood at work compared to positive perceptions. Participants reporting a greater intensity of mood swings had consistently higher odds of reporting negative perceptions of mood at work. Finally, age, current contraceptive use, MDQ behavior change and concentration subscales were all significantly associated with perceptions of level of interest in work, with distinct threshold-specific patterns identified through the Bayesian PPO models. Age and contraceptive use were significantly associated only at the higher threshold, with older females and those that reported use of contraceptives showing lower odds of reporting positive perceptions in interest in work compared to neutral perceptions. The MDQ behavior change and concentration subscales were significant only at the lower threshold, such that participants with higher subscale scores had higher odds of reporting negative perceptions of interest at work compared to neutral perceptions. Exos employment status was not significantly associated with any outcome in the CLMMs. However, in the Bayesian models for coworker relationships and efficiency, Exos employees had higher odds of reporting neutral (vs. negative) perceptions. These associations were detected only in the Bayesian framework, which allows for threshold-specific effects by relaxing the proportional odds assumption. Full results, including estimates of threshold-specific effects for PPO models, are provided in Additional File 1, Appendix D.

Nearly half (49% and 50.6%) of the overall and non-Exos participants, respectively, reported feeling ‘very to extremely’ equipped to manage their hormonal-related symptoms. Most participants (94.4%) reported that they would or might be willing to participate in menstrual health programming that addresses improving overall well-being. Similarly, 95.7% reported that they would or might be interested in learning more about the menstrual cycle with the greatest interest in physical performance, energy management, sleep quality, and mood. Most participants in the overall and non-Exos samples (69% and 68%), respectively, reported being ‘very to extremely’ comfortable receiving menstrual health educational content from a woman. Similarly, 55% and 53% of the overall sample reported being ‘very to extremely’ comfortable receiving menstrual health educational content from peers their age or older than them, respectively.

## Discussion

This study reports novel findings on the impact of hormonal-related symptoms on perceived work productivity across cyclical hormone fluctuations and the willingness to engage in menstrual health programs among females of reproductive age working in the U.S. The findings demonstrate that hormonal-related symptoms are experienced throughout all hormonal phases, with symptom severity varying by demographics or menstrual experiences. Despite most participants reporting menstrual pain, nearly half felt ‘very to extremely’ equipped to manage their hormonal-related symptoms. However, perceptions of work-related productivity fluctuated across hormonal phases, being significantly more negative during the bleed and pre-bleed phases and more positive during the late follicular and early luteal phases. After controlling for covariates, higher intensities of hormonal-related disturbances indicated more negative perceptions of productivity. Despite the impact, very few participants reported taking time off from work due to their menstrual cycle. Most participants expressed willingness to engage in menstrual health programming, although their employers often did not provide menstrual-related benefits. Participants preferred learning about menstrual health information from females of similar age or older.

Consistent with earlier studies, our findings showed that most participants in the overall sample reported experiencing menstrual-related pain (91% and 89.5%, respectively) [3, 4]. This was expected as dysmenorrhea is considered highly prevalent [3, 4]. The top two hormonal-related symptoms, in the overall sample, during the bleed phase were fatigue and cramps (uterine or pelvic), which aligns with the broader literature [4, 13, 26–28]. Our participants experienced multiple symptoms throughout each hormonal phase, consistent with the limited work evaluating menstrual symptom cluster analyses [29–31]. This is crucial as previous work often assessed single symptoms’ impact on productivity loss, potentially missing the real-world experience of concurrent symptoms.

Results from the sensitivity analysis conducted to account for 40% of participants being Exos employees, showed that there were a greater number of symptoms with average intensities that differed significantly across hormonal phases in the overall sample compared to the non-Exos sample. The smaller number of statistically significant results in the non-Exos group may reflect reduced statistical power due to the smaller sample size, rather than a true absence of group differences. In fact, most symptom intensities differed by only ± 0.10 between the overall and non-Exos samples, suggesting the patterns are largely consistent across groups. An additional explanation for this is that the average age of Exos employees was nearly three years younger than that of non-Exos employees. Research suggests that hormone-related symptoms may be more prevalent and severe in younger females compared to their older peers during menstruation [6, 32]. These findings were consistent with other findings from the current study that show age related differences across MDQ subscale scores in which participants 22–29 years had higher scores across all MDQ subscale scores compared to their older peers.

The understanding of menstrual-related symptoms experienced during the intermenstrual phase is limited [33–36]. In the current study, twenty-one bleed-phase related disturbances, including cramps, were reported by at least 25% of participants during the intermenstrual phase. These disturbances could be due to ovulation pain, experienced by up to 40% of females, or premenstrual syndrome (PMS), which can appear anytime during the luteal phase [36, 37]. In a study of over 3,000 working women in Japan, Ozeki et al. [36] found that utilizing a PMS education checklist led to reduced intermenstrual MDQ scores [-8.44 points (95% CI: -14.73 to -2.15 points)], by follow-up, in participants with moderate-to-severe PMS symptoms who sought medical attention. The purpose of the PMS education checklist was to inform and create awareness of PMS. The checklist included eleven common PMS symptoms and advised participants to seek medical consultation if two or more symptoms were checked from the list. The reduction in MDQ scores among participants who sought medical attention was believed to have been attributed to the fact that they sought the assistance that resulted in the alleviation of their symptoms [36]. Most participants did not seek medical attention; therefore, the authors suggested that other lifestyle programs would be helpful for those uncomfortable seeking care [36].

Grandi et al. [35] investigated the impact of pelvic pain on quality of life in 300 patients and reported that intermenstrual pain was statistically associated with reduced quality of life and depressive mood. Our findings demonstrated that anxiety was the most prevalent (51.1%) and second most prevalent (47.5%) symptom during the remainder of the current cycle, in the overall and non-Exos samples, respectively. This is consistent with studies showing heightened anxiety in the luteal phase underscoring the impact of cyclical hormone fluctuations on female mental health [33, 38]. Research shows that mental health, specifically depression and anxiety, negatively impacts work-related productivity [39]. A systematic review found moderate evidence for the value of mental health interventions on work-related outcomes with the greatest support for programs intended to improve mental and physical health [40].

In the present study, participants reported a greater degree of negative perceptions across all work-related productivity measures during the pre-bleed and bleed phases and a greater degree of positive perceptions during the late follicular and early luteal phases. Ponzo et al. revealed that participants reported moderate-to-severe negative impact of their menstrual cycle on concentration at work (77.2%), efficiency (68.3%), energy levels (89.3%), relationships with coworkers (39.0%), interest in their own work (71.6%), and mood (86.9%) [13]. In comparison, this study showed lower reports of ‘somewhat to extremely’ negative impact on concentration (53.2% and 53.8%), efficiency (48.9% and 51.1%), energy (81.4% and 80.7%), relationships with coworkers (19.9% and 24.2%), levels of interest in work (42.4% and 44.8%), and mood at work (58.8% and 61.4%) during the bleed-phase in the overall and non-Exos samples, respectively. The percent differences between studies is likely attributed to the modifications made to these measures in this study to better understand the directionality of hormonal phase impact on work-related productivity. Despite these differences, the rank order of negatively impacted work-related productivity measures across the two studies is nearly identical. Findings from the current study also showed that perceptions of relationships with coworkers were more negative in Phase 4 compared to Phase 1. These findings are consistent with a study in 186 academic females in Egypt in which half of participants reported impairments in their relationships with their coworkers as a result of their premenstrual symptoms [41]. Coworker relationships may be disturbed due to premenstrual symptoms given that individuals spend a substantial portion of their waking hours interacting with their coworkers, either in person or remotely. Collectively, these findings support previous work that suggests that the menstrual cycle has a significant effect on work-related productivity [5–14].

To our knowledge, this was the first study to assess the directionality of multiple work-related productivity areas across cyclical hormone phases and the relationship of self-reported hormonal-related symptoms to perceived work-related productivity. Generally, the symptom-related findings were largely consistent with theoretical expectations. For example, more intense bursts of energy were associated with greater perceptions of energy, independent of other covariates. Similarly, we found that many hormonal-related disturbances had significant inverse relationships with measures of perceived work-related productivity independent of other covariates. Participants were more likely to report more positive perceptions of work-related productivity during the late follicular and early luteal phases than the bleed-phase. However, more granularly, differences in distributions were observed in perceived efficiency, energy, and mood at work (*p* ≤ 0.05) between the late follicular and early luteal phases. Future work-related productivity research should include the intermenstrual phase and explore separating this phase into two independent phases (late follicular and early luteal).

Two variables, mood swings and contraceptive use, modeled with proportional odds in both the CLMM and Bayesian frameworks were significant only in the Bayesian models, likely due to the combined effects of the conservative Bonferroni adjustment and the Bayesian approach’s greater flexibility in estimating uncertainty. Other variables, including age, Exos employment, contraceptive use, and several MDQ subscale scores, modeled with non-proportional odds, were significant at one or both thresholds within the Bayesian framework but not in the CLMMs. For example, in both the relationships with coworkers and efficiency Bayesian models, Exos employment status was significantly associated with reporting higher odds of neutral perceptions relative to negative perceptions. These localized effects could be attributed to response bias such that Exos employees may have felt less comfortable reporting negatively about their ability to maintain relationships with their coworkers or perform efficiently since the study was conducted by their employer. However, given that Exos is a human performance company with an emphasis on well-being and performance, these findings may reflect contextual or cultural influences, such as a workplace environment that supports employees in ways that buffer against negative perceptions or promote more neutral evaluations of relationships and efficiency. Regardless of explanation, these findings reinforce the value of threshold-specific modeling in uncovering subtle associations that may otherwise go undetected.

Consistent with the literature, this study demonstrated that age, BMI, and heavy bleeding experience are significantly associated with hormonal-related symptom severity (*p* ≤ 0.05) [3, 6, 11, 32, 42–49]. However, after controlling for potential confounders, BMI and heavy bleeding experience were not significantly associated with perceptions of work-related productivity. Age had a significant inverse association across several perceptions of work-related productivity outcomes, including perceptions of concentration, relationships with coworkers, and efficiency using the frequentist CLMMs. The association between age and relationship with coworkers was maintained as a general effect in the Bayesian framework; however, the inverse relationship between age and concentration, and efficiency were significant only at the higher threshold, such that older participants had lower odds of reporting the most positive perceptions in these work-related areas of productivity. Age also showed a significant inverse relationship with energy, mood at work, and level of interest in work in the higher threshold of the Bayesian framework but was not significant in the CLMMs. To our knowledge, there are currently no studies that assess the relationship between older premenopausal females and perceptions of work-related concentration, energy, efficiency, mood, level of interest in work, or relationships with coworkers. However, many symptoms experienced during premenopause are consistent with perimenopausal symptoms; therefore, one possibility for these findings is that older females in this study may be experiencing early symptoms related to the perimenopausal transition that may be impacting their perceptions of work-related productivity in these areas [50]. For example, research has shown that as females age, specifically from midlife on, they experience hormonal shifts that impact energy, fatigue, concentration, and mood changes specifically as they approach and enter perimenopause [51–53]. Similarly, older females are more likely to practice work-family balance, experience age-related work discrimination, and intergenerational differences with their younger peers which may impact their ability to create and maintain relationships with their coworkers [54, 55].

Contraceptive use showed a significant inverse relationship with perceptions of concentration in both the frequentist cumulative link models and the Bayesian models, and efficiency and level of interest in work in the Bayesian models. Contraceptive use was only significant in the higher threshold of the Bayesian models such that participants that reported using contraceptives had higher odds of reporting more neutral perceptions of level of interest in work compared to positive perceptions. While there are individual studies that support the finding that contraceptive use is inversely associated with cognitive performance [56, 57], a recent systematic review by Gurvich et al. [58] has reported that the relationship between contraceptive use and cognitive performance is inconsistent. Also, in the present analysis, the variable contraceptive use consisted of various hormonal and non-hormonal contraceptives. As such, it is unclear which forms of contraceptives are driving these relationships. Collectively, these findings suggest that hormonal-related symptom severity may have a stronger association with perceptions of work-related productivity than demographics.

Variables such as decreased efficiency and multiple MDQ subscales including, MDQ concentration, MDQ arousal, and MDQ negative showed U-shaped patterns with perceptions of work-related productivity in Bayesian models such that higher symptom or MDQ subscale intensities were linked to both lower odds of neutral responses and higher odds of either negative or positive outcomes. This suggests that broader symptom clusters, consisting of numerous heterogeneous symptoms, may be associated with more polarized self-perceptions of work-related productivity outcomes. Alternatively, since the MDQ subscales consist of numerous individual symptoms, higher scores may be driven by varying symptoms across participants therefore it is unclear which symptoms are specifically driving these U-shaped relationships. However, consistent with the Yerkes-Dodson law that indicates a U-shaped relationship between psychological arousal and job performance, this may highlight a threshold of optimal versus excessive impact of symptom intensity on perceptions of work-related productivity [59]. This may also suggest that some symptoms may either enhance or disrupt functioning in an individual or reinforce the importance of relevant symptom clustering based on their relationship to a given work-related productivity outcome.

In general, the Bayesian models produced more conservative and stable estimates than the CLMMs, which showed signs of inflation in bootstrap resampling, likely due to sparse or imbalanced outcome categories in Phases 2 and 3. These Bayesian estimates offered a useful comparison point for understanding the strength and direction of phase effects, particularly where CLMM estimates may have overstated the association. Strong associations between Phases 2 and 3 and improved work-related productivity outcomes were consistently identified across both modeling approaches, reinforcing the robustness of this effect despite differences in model specification. Taken together, these findings highlight the value of combining frequentist and Bayesian methods in modeling ordinal data. The adjacent category structure, paired with PPO modeling, enabled detection of subtle or non-proportional effects that might otherwise remain hidden.

Similar to this study’s findings (13.2% and 13.4%) for the overall and non-Exos samples, respectively, a cohort of 32,749 Dutch women reported that 19.3% of participants reported missed days of work or school due to their menstrual cycle [18]. Despite the presence and severity of hormonal-related symptoms and the perceived impact on their productivity, most of our participants refrained from taking days off from work due to their menstrual cycle. In a study of 1,800 U.S. females, 5.4% of participants reported having access to menstrual-related benefits; the majority (75.6%) of participants who did not receive benefits expressed a desire [13]. These results are comparable to our sample, where 4.6% of all participants reported that their employer offered menstrual health benefits or wellness programs, with the majority reporting that menstrual health programming that addresses improving overall well-being would or might be something in which they would participate.

The current study uniquely sought to understand the receptivity to menstrual health programmatic content. Nearly all (95.7%; *N* = 369) participants showed interest in learning more about menstrual health, with 62% (*N* = 371) tracking their cycle ‘very often to always’. These percentages were consistent in the non-Exos sample. Despite nearly half feeling ‘very to extremely’ well equipped to manage their hormonal-related symptoms, the current sample expressed a great interest in content that would optimize their cycle and mitigate commonly reported hormonal-related symptoms like physical performance, energy management, sleep quality, and mood. Further, we sought to understand the receptivity to menstrual-programming delivery and found that participants were most comfortable receiving menstrual health programming from other females who were comparable in age or older peers.

Digital health apps and lifestyle programs, including physical activity and education-based content, have shown improvements in worker quality of life and productivity, and reduce the severity of some menstrual-related symptoms [13, 60, 61]. Established lifestyle programs can serve as a base for employer-sponsored menstrual health program development, aiming to optimize productivity during the late follicular and early luteal phases and minimize negative impacts during the pre-bleed and bleed phases. Other considerations should include stratifiers based on demographics, such as age, BMI, ethnicity, and race, or menstrual-related experiences and conditions such as heavy bleeding, PMS, or dysmenorrhea for more targeted alleviation of symptoms. However, programming against the presence and severity of specific or clusters of symptoms such as difficulty concentrating, decreased efficiency, mood swings, fatigue, and anxiety, may have a greater impact on work-related productivity.

This study’s strengths include a unique sample of U.S. working females, the evaluation of a wide range of affective and physical hormonal-related symptoms, and the inclusion of multiple work-related productivity measures assessed for bi-directional impact across four cyclical hormone fluctuation phases. Research investigating the lived menstrual cycle experience by working U.S. females is limited, as most related studies have primarily been conducted elsewhere [9, 13]. Environmental, geographical, and lifestyle exposures may have implications for menstrual cycle function and the risk of developing premenstrual disorders [62, 63]; therefore, research conducted outside of the United States may not best represent the U.S. female population [18, 22, 36, 64, 65].

Limitations of the current work include cross-sectional associations, the use of self-reported subjective data, modifications to Flo App’s Menstrual Cycle-Related Work Productivity Questionnaire [13] without pilot validation data, potential response bias, potential commercial interest, and the limited representation of racial diversity. Nearly 80% of the study participants were White. It is well established that racial disparities exist for reproductive health outcomes, menstrual care access, period poverty, and menstrual health issues [66–68]. It is also recognized that menstrual experiences differ by race and ethnicity, including the age of menarche, menstrual cycle length and variability, and in turn the risk of developing premenstrual risks during adulthood [69, 70]. Given the lack of racial diversity in this study, this limits the generalizability of these results and warrants the need for future studies to include a more diversified sample across races to more robustly evaluate these relationships. Given that 40% of participants were employees of the study funding organization this introduces the potential for response bias and therefore may limit the generalizability of the study findings. To ensure scientific integrity and minimize the potential for bias, participation in this study was completely voluntary, the study objectives were unrelated to employment positions and duties, and employment was in no way impacted based on someone’s election to participate in the study. Future research should aim to conduct large studies consisting of females across more diverse industries and organizations to confirm and expand upon these findings. Lastly, while every effort was made to ensure the objectivity and integrity of the research, it is important to acknowledge that, given the authors’ affiliation with the funding agency, either through employment or contract, this study may be subject to potential commercial influence. Readers should interpret the findings with consideration in mind.

Prospective longitudinal studies are needed to validate these findings. Future work should leverage objective work-related productivity measures and utilize cluster analyses to understand the personalized nature of hormonal-related symptoms. Due to survey tool limitations in the current study, the intermenstrual phase definition differed between the MDQ and the work-related productivity assessment. Future studies should consider parsing out the experience of MDQ symptoms by late follicular and early luteal phases. Lastly, although multiple comparison corrections were applied within each analytically distinct group, the study involved many comparisons across models and outcomes, and findings should be interpreted with an awareness of this broader multiplicity and validated in future research.

## Conclusions

Cyclical hormone fluctuations may be indicative of perceived work-related productivity. Although working U.S. females highly desire menstrual health programming, few have access to such resources. Recommended program elements that should be considered when developing, testing, and validating such programs include, but are not limited to, menstrual-related education, programming for mental and physical health, program delivery, and dissemination. Menstrual-related education should prioritize the physiology of cyclical hormone fluctuations, promote self-awareness of symptom recognition including symptoms affiliated with specific cyclical hormone phases that may be advantageous for collaborative or individual-based work, and embolden self-advocation for medical treatment when necessary. Based on the current study findings, programs may best be received if delivered by females of similar age or older than the target audience. Lastly, to broaden program dissemination, the development of in-person and digital experiences would provide females with the ability to benefit from these programs if they work remotely or choose to work remotely to accommodate their hormone-related symptoms. Similarly, digital experiences will allow females the ability for continuity of program experience if they work in a hybrid environment. For in-person programs, room and facility accommodations should be inviting, safe, and private. Similarly, program size for in-person experience should be evaluated for program effectiveness and adherence. However, future research is needed to test these recommendations in practice, and to more robustly understand symptom profiles and the relationship between hormonal-related symptoms and work-related productivity. These insights will aid in designing employer-sponsored menstrual health programming to mitigate hormonal-related symptoms and optimize performance throughout cyclical hormone fluctuations in working U.S. females.

## Electronic supplementary material

Below is the link to the electronic supplementary material.

**Additional file 1: Appendix A:** Exos Female Physiology Questionnaire. **Appendix B:** Education and work-related statistics of 372 full-time females of reproductive age in the United States. **Appendix C:** Menstrual Distress Questionnaire by cyclical hormone phase for non-Exos employees. **Appendix D:** Model estimates from Bayesian adjacent category ordinal models for perceptions of work-related productivity outcomes

## Data Availability

The participants in this study did not give written consent for their data to be shared publicly. Due to participant privacy, supporting data are not available. Any additional information required to reproduce the results reported herein can be requested by the corresponding author, DMR, and evaluated based on the criteria for access to confidential data.

## Abbreviations

U.S.: United States
EFPQ: Exos Female Physiology Questionnaire
IRB: Institutional review board
MDQ: Menstrual Distress Questionnaire
BMI: Body mass index
FDR: False discovery rate
CLMM: Cumulative link mixed models
AIC: Akaike information criterion
CI: Confidence interval
OR: Odds ratio
PPO: Partial proportional odds
LOO: Leave-one-out cross-validation
CrI: Credible interval
SD: Standard deviation
Prev: Prevalence
Int: Average intensity
PMS: Premenstrual syndrome
