# Supplementary material for: A survey assessing the impact of symptoms related to the menstrual cycle and perceptions of workplace productivity: considerations for employer-sponsored menstrual health programs
Source: BMC Womens Health. 2025 Aug 30;25:418. doi: 10.1186/s12905-025-03833-w (PMC12398178; doi:10.1186/s12905-025-03833-w)
Supplement: Supplementary file 1 — Additional file 1: Appendix A: Exos Female Physiology Questionnaire. Appendix B: Education and work-related statistics of 372 full-time females of reproductive age in the United States. Appendix C: Menstrual Distress Questionnaire by cyclical hormone phase for non-Exos employees. Appendix D: Model estimates from Bayesian adjacent category ordinal models for perceptions of work-related productivity outcomes [file 12905_2025_3833_MOESM1_ESM.pdf]

Appendix A. Exos Female Physiology Questionnaire

This information will be used for analysis purposes only. This means that all data from this questionnaire will be de-identified, remain anonymous, and only reported in aggregate. Please answer all questions honestly and to the best of your ability.

This questionnaire should take approximately 20 minutes to complete; however, the time to complete may vary by participant.

Note: Responses that are marked exclusionary concluded participation in the study, unless otherwise stated. Ineligible participants were rerouted to a study end message where they were notified of their ineligibility.

ABOUT YOU

The following questions ask for information about you.

1. Do you currently live in the United States? (Excluding US territories)
   Yes
   No *[exclusionary]*

2. What state do you currently reside in? ____________________

3. Are you currently an Exos employee (either full or part-time)?
   Yes
   No *[exclusionary – Phase 1 only]*

4. Are you fluent in English?
   Yes
   No *[exclusionary]*

5. Based on the state or territory you currently reside in, are you legally considered an adult?

   Nebraska and Alabama - greater than or equal to 19 years old
   Mississippi - greater than or equal to 21 years old
   Remaining US states (excluding US territories) - greater than or equal to 18 years old
   Yes
   No *[exclusionary]*

6. Birth Month_______________

7. Birth Year________________

8. Biological sex (at birth)
   Male *[exclusionary]*
   Female
   Prefer not to say *[exclusionary]*
   Other *[exclusionary]*

9. What is your gender identity?
   Man *[exclusionary]*
   Woman

Non-Binary Person
Third Gender
Prefer not to say *[exclusionary]*
Other *[exclusionary]*

10. Race [Please select all that apply]
    American Indian or Alaskan Native
    Asian
    Black or African American
    Native Hawaiian or Other Pacific Islander
    White
    Prefer not to say
    Other

11. Ethnicity
    Hispanic or Latino
    Not Hispanic or Latino
    Prefer not to say

12. Height (in feet and inches)______________

13. Current Weight (in lbs.) ____________

14. Highest educational level completed?
    High school
    2-year college
    4-year college
    Master's degree
    Ph.D. or other doctorate degree
    Prefer not to say
    Other ____________

15. Are you a full-time employee?
    Yes
    No
    Other, please explain ____________
    Prefer not to say

16. What is your current work environment?
    Onsite (e.g. office)
    Remote
    Hybrid
    I do not currently have a job
    Prefer not to say

Modified from Source: Menstrual Cycle-Related Work Productivity Questionnaire [developed by Flo Health app ("Flo App")]
Ponzo S, Wickham A, Bamford R, Radovic T, Zhaunova L, Peven K, et al. Menstrual cycle-associated symptoms and workplace productivity in US employees: A cross-sectional survey of users of the Flo mobile phone app. DIGITAL HEALTH. 2022 Jan;8:205520762211458.

17. What industry do you currently work in? *[do not display if current environment = I do not currently have a job]*

    Accommodation and Food Services
    Administrative and Support and Waste Management and Remediation Services
    Agriculture, Forestry, Fishing and Hunting
    Arts, Entertainment, and Recreation
    Construction
    Educational Services
    Finance and Insurance
    Health Care and Social Assistance
    Information
    Management of Companies and Enterprises
    Manufacturing
    Mining, Quarrying, and Oil and Gas Extraction
    Professional, Scientific, and Technical Services
    Public Administration
    Real Estate and Rental and Leasing
    Retail Trade
    Transportation and Warehousing
    Utilities
    Wholesale Trade
    Other Services

18. Which of the following best describes the size of your company, in total employees? *[do not display if current environment = I do not currently have a job]*

    0-50
    51-250
    251-500
    501-1000
    1001-1500
    1501-3000
    3001-5000
    5000+
    Prefer not to say

Source: Menstrual Cycle-Related Work Productivity Questionnaire [developed by Flo Health app ("Flo App")]
Ponzo S, Wickham A, Bamford R, Radovic T, Zhaunova L, Peven K, et al. Menstrual cycle-associated symptoms and workplace productivity in US employees: A cross-sectional survey of users of the Flo mobile phone app. DIGITAL HEALTH. 2022 Jan;8:205520762211458.

19. What is your current work role? *[do not display if current environment = I do not currently have a job]*

    Clerical
    Entry-level
    Executive management
    Laborer
    Manufacturing position
    Medical professional
    Middle management
    Professional

Service worker
Skilled trades
Supervisor
Technical
Prefer not to say

Source: Menstrual Cycle-Related Work Productivity Questionnaire [developed by Flo Health app ("Flo App")]
Ponzo S, Wickham A, Bamford R, Radovic T, Zhaunova L, Peven K, et al. Menstrual cycle-associated symptoms and workplace productivity in US employees: A cross-sectional survey of users of the Flo mobile phone app. DIGITAL HEALTH. 2022 Jan;8:205520762211458.

MENSTRUAL FUNCTION
Mark the response that most accurately describes your situation.

20. How old were you when you had your first period?

11 years or younger
12-14 years
15 years or older
I don't remember
I have never menstruated *[exclusionary]*
Prefer not to say

Source: LEAF-Q
*Melin A, Tornberg ÅB, Skouby S, et al. The LEAF questionnaire: a screening tool for the identification of female athletes at risk for the female athlete triad. British Journal of Sports Medicine 2014;**48:**540-545.*

21. How would you describe your current menstrual status?

I am of reproductive age
I am in the perimenopause/menopause transition (changes in periods, but have not gone 12 months in a row without a period)
I am unsure if I am perimenopausal/menopausal
I have gone through menopause (experienced 12 months or longer without a period) *[exclusionary -proceed to Q22]*
Prefer not to say *[exclusionary]*

22. Have you had a hysterectomy? *[final question for menstrual status = I have gone through menopause]*

Yes *[exclusionary]*
No
Prefer not to say *[exclusionary]*

23. When was your last period?

0-4 weeks ago
1-2 months ago
3-4 months ago *[exclusionary]*
5-6 months ago *[exclusionary]*
7 - 12 months ago *[exclusionary]*
12 months ago or more *[exclusionary]*
I'm pregnant or breastfeeding and DO NOT currently menstruate *[exclusionary]*

Prefer not to say

USE OF CONTRACEPTIVES

You are being asked the following questions as there are different ways to regulate the female physiology.

Mark the response that most accurately describes your situation.

24. Which of the following contraceptives do you currently use? [Please select all that apply]

    Pill
    Implant
    IUD (hormonal or non-hormonal)
    Shot
    None of the above
    Prefer not to say

MENSTRUAL SYMPTOMS

25. Have you missed any work in the past 6 months as a result of your menstrual cycle?

    Yes
    No
    Unsure
    Prefer not to say

26. How many days of work have you missed in the past 6 months as a result of your menstrual cycle? *[do not display if missed any work in the past 6 months= "No" or "Unsure" or "Prefer not to say"]*

____

27. How well equipped do you feel to manage symptoms associated with your menstrual cycle?

    Not at all
    Slightly
    Moderate
    Very
    Extremely

28. Do you or have you experienced menstrual pain?

    Yes
    No
    Prefer not to say

MENSTRUAL SYMPTOMS – MENSTRUAL DISTRESS QUESTIONNAIRE (MDQ)

29. Please reference the Menstrual Distress Questionnaire (MDQ), developed by Moos to view this survey. Participants rated 47 symptoms, across 3 time frames, on a 5-point likert scale (no experience of a symptom, mild, moderate, strong, disabling). Participants could select "prefer not to say" if they did not wish to answer the question.

Source: Menstrual Distress Questionnaire
Moos RH. The development of a menstrual distress questionnaire. Psychosom Med. 1968;30(6):853–67.

MENSTRUAL PHASE EXPERIENCE
The following questions ask about how you feel and the impact you may experience during each of the four menstrual phases.

Note: A visual of the menstrual cycle phase division diagram, developed by Solli et al. was provided to participants as reference.
Source: Solli G, Sandbakk S, Noordhof D, Ihalainen J, Sandbakk O. Changes in Self-Reported Physical Fitness, Performance, and Side Effects Across the Phases of the Menstrual Cycle Among Competitive Endurance Athletes. International journal of sports physiology and performance. 2020 Sep 21;15.

NOTE: The following questions repeat for each phase 1-4

Questions 30-35 were modified from source: Menstrual Cycle-Related Work Productivity Questionnaire [developed by Flo Health app ("Flo App")]
Ponzo S, Wickham A, Bamford R, Radovic T, Zhaunova L, Peven K, et al. Menstrual cycle-associated symptoms and workplace productivity in US employees: A cross-sectional survey of users of the Flo mobile phone app. DIGITAL HEALTH. 2022 Jan;8:205520762211458.

30. The following questions pertain to **Phase** _____ of your menstrual cycle:
    How much does this phase of your menstrual cycle impact your: **concentration**
    - Extremely negative impact
    - Somewhat negative impact
    - Neither negative nor positive impact
    - Somewhat positive impact
    - Extremely positive impact

31. The following questions pertain to **Phase** _____ of your menstrual cycle: How much does this phase of your menstrual cycle impact your: **efficiency**
    Extremely negative impact
    Somewhat negative impact
    Neither negative nor positive impact
    Somewhat positive impact
    Extremely positive impact

32. The following questions pertain to **Phase** _____ of your menstrual cycle:
    How much does this phase of your menstrual cycle impact your: **energy levels**
    Extremely negative impact
    Somewhat negative impact
    Neither negative nor positive impact
    Somewhat positive impact
    Extremely positive impact

33. The following questions pertain to **Phase** _____ of your menstrual cycle:
    How much does this phase of your menstrual cycle impact your: **relationship with coworkers**
    Extremely negative impact
    Somewhat negative impact
    Neither negative nor positive impact
    Somewhat positive impact
    Extremely positive impact

34. The following questions pertain to **Phase** _____ of your menstrual cycle:
    How much does this phase of your menstrual cycle impact your: **level of interest in your work**
    Extremely negative impact
    Somewhat negative impact
    Neither negative nor positive impact
    Somewhat positive impact
    Extremely positive impact

35. The following questions pertain to **Phase** _____ of your menstrual cycle:
    How much does this phase of your menstrual cycle impact your: **mood at work**
    Extremely negative impact
    Somewhat negative impact
    Neither negative nor positive impact
    Somewhat positive impact
    Extremely positive impact

MENSTRUAL PRACTICE

The following questions ask about your current menstrual practices.

36. How often do you track your cycle?
    Never
    Rarely
    Sometimes

Very Often
Always
Prefer not to say

MENSTRUAL WILLINGNESS & ACCESSIBILITY
The following questions ask about your willingness and preferences around cycle-based programming and education. Please select the responses that best represent you. There are no right or wrong answers.

37. How comfortable are you receiving **menstrual cycle-based educational content** from:

**Source**	**Not at all**	**Slightly**	**Moderately**	**Very**	**Extremely**
Peers your own age					
Peers younger than you					
Peers older than you					
A woman					
A man					

38. Does your employer offer any benefits or wellness programs related to your menstrual cycle?
    Yes
    No
    I don't know
    Prefer not to say

39. Would a cycle-based program that addressed improving your overall well-being be something you would participate in?
    Yes
    No
    Maybe
    Prefer not to say

40. Would you be interested in learning more about how the menstrual cycle relates to any of the following areas?
    Physical performance
    Work performance
    Energy management
    Sleep quality
    Menstrual cycle physiology
    Mood
    Social connection and relationships

Yes
No *[end survey]*
Maybe
Prefer not to say

41. Please rank the following areas by dragging each in the order that you are most interested in learning more about.
[1- Most interested; 7- Least interested].
    ______ Physical performance
    ______ Work performance
    ______ Energy management
    ______ Sleep quality
    ______ Menstrual cycle physiology
    ______ Mood
    ______ Social connection and relationships

--END SURVEY--

Appendix B. Education and work-related statistics of 372 full-time females of reproductive age in the United States

	n	*%*
**Highest Education Level Completed**		
High School	6	1.6
2 - year college	13	3.5
4 - year college	189	50.8
Master's degree	144	38.7
Doctor or professional degree	19	5.1
Other	0	0.0
Prefer not to say	1	0.3
**Company Size**		
0-50	62	16.7
51-250	30	8.1
251-500	25	6.7
501-1000	34	9.1
1001-1500	20	5.4
1501-3000	56	15.1
3001-5000	43	11.6
5000+	83	22.3
Prefer not to say	19	5.1
**Industry**		
Other Services	134	36.0
Health Care and Social Assistance	96	25.8
Professional, Scientific, and Technical Services	33	8.9
Educational Services	25	6.7
Arts, Entertainment, and Recreation	21	5.6
Finance and Insurance	17	4.6
Management of Companies and Enterprises	12	3.2
Manufacturing	8	2.2
Information	8	2.2
Retail Trade	6	1.6
Agriculture, Forestry, Fishing and Hunting	5	1.3
Real Estate and Rental and Leasing	3	0.8
Construction	2	0.5
Prefer not to say	2	0.5
**Work Role**		
Professional	114	30.6
Middle Management	87	23.4
Entry-Level	50	13.4
Medical Professional	40	10.8
Executive Management	23	6.2
Supervisor	17	4.6
Technical	8	2.4
Service Worker	7	1.9
Skilled Trades	6	1.6
Manufacturing Position	2	0.5
Clerical	1	0.3
Prefer not to say	16	4.3

Appendix C. Menstrual Distress Questionnaire by cyclical hormone phase for non-Exos employees

Symptoms	Bleed-phase (N = 223)			Pre-bleed Phase (N = 223)			Intermenstrual (N = 223)			Significance Level			
	n	Prev	Int	n	Prev	Int	n	Prev	Int	Comparison Across Phases[1]	Bleed-phase vs. Pre-bleed Phase[2]	Bleed-phase vs. Intermenstrual[2]	Pre-bleed Phase vs. Intermenstrual[2]
Fatigue	184	86.5%	1.77	136	61.0%	1.49	97	43.5%	1.51	**	**	*	
Cramps (uterine or pelvic)	192	86.1%	1.84	87	39.0%	1.37	57	25.6%	1.49	**	***	*	
Change in eating habits	185	83.0%	1.85	139	62.3%	1.67	93	48.9%	1.43	**		***	*
Irritability	169	75.8%	1.87	125	56.1%	1.79	78	35.8%	1.45	**		***	**
Mood swings	164	73.5%	1.74	125	56.1%	1.66	67	32.0%	1.51				
Anxiety	162	72.6%	1.93	134	60.1%	1.76	106	51.1%	1.50	**		***	*
General aches and pains	153	68.6%	1.55	84	37.7%	1.36	71	35.8%	1.28	*		*	
Affectionate	140	62.8%	1.65	103	46.2%	1.61	114	57.0%	1.47				
Backache	134	60.1%	1.82	80	35.9%	1.48	64	30.6%	1.50	*	*		
Headache	129	57.8%	1.68	78	35.0%	1.54	62	32.5%	1.39				
Decreased efficiency	137	61.4%	1.63	72	32.3%	1.33	46	20.7%	1.39	*	**		
Depression (feeling sad or blue)	127	57.0%	1.72	105	47.1%	1.63	60	28.8%	1.52				
Crying	125	56.1%	1.77	106	47.5%	1.66	52	25.2%	1.62				
Painful breasts/chests	120	53.8%	1.46	92	41.3%	1.42	34	16.7%	1.35				
Avoid social activities	126	56.5%	1.70	73	32.7%	1.64	57	26.1%	1.51				
Weight gain	129	57.8%	1.40	82	36.8%	1.34	45	20.4%	1.42				
Difficulty concentrating	122	54.7%	1.58	82	37.1%	1.51	49	25.5%	1.41				
Feelings of well-being	107	48.0%	1.60	85	40.3%	1.65	98	44.9%	1.78				
Swelling (abdomen, breasts, or ankles)	103	46.2%	1.52	74	38.4%	1.34	30	18.0%	1.30				
Easily distracted	103	46.2%	1.56	71	34.7%	1.38	56	25.1%	1.30				
Insomnia (sleeplessness)	107	48.0%	1.59	70	32.8%	1.46	56	25.1%	1.41				
Muscle stiffness	86	38.6%	1.49	59	30.6%	1.31	53	23.8%	1.30				
Burst of energy or activity	84	37.7%	1.68	68	34.7%	1.54	93	41.7%	1.60				
Takes naps, stay in bed	96	43.0%	1.50	53	29.6%	1.51	43	19.3%	1.33				
Tension	94	42.2%	1.41	53	29.6%	1.36	45	20.2%	1.31				
Skin troubles - spots	90	40.4%	1.43	79	35.4%	1.44	57	25.6%	1.39				
Lowered school or work performance	44	19.7%	1.32	24	10.8%	1.33	19	8.5%	1.37				

Forgetfulness	37	16.6%	1.51	31	13.9%	1.45	20	9.0%	1.40
Restlessness	45	20.2%	1.31	31	13.9%	1.55	39	17.5%	1.54
Loneliness	38	17.0%	1.45	17	7.6	1.47	15	6.7%	1.33
Excitement	38	17.0%	1.24	19	8.5%	1.42	20	9.0%	1.20
Accident-prone (cut finger, break dish, etc.)	33	14.8%	1.58	23	10.3%	1.49	17	7.6%	1.18
Dizziness, faintness	33	14.8%	1.61	19	8.5%	1.39	14	6.3%	1.50
Hot flashes	33	14.8%	1.79	20	9.0%	1.52	21	9.4%	1.48
Confusion	30	13.5%	1.47	15	6.7%	1.38	14	6.3%	1.21
Lowered motor coordination	31	13.9%	1.58	16	7.2%	1.34	10	4.5%	1.40
Lowered judgment	44	19.7%	1.32	24	10.8%	1.45	19	8.5%	1.37
Orderliness	37	16.6%	1.51	31	13.9%	1.55	20	9.0%	1.40
Blind spots, fuzzy vision	45	20.2%	1.31	31	13.9%	1.46	39	17.5%	1.54
Nausea or vomiting	38	17.0%	1.45	17	7.6%	1.46	15	6.7%	1.33
Heart pounding	38	17.0%	1.24	19	8.5%	1.38	20	9.0%	1.20
Cold sweats	33	14.8%	1.58	23	10.3%	1.61	17	7.6%	1.18
Feelings of suffocation	33	14.8%	1.61	19	8.5%	1.54	14	6.3%	1.50
Chest pains	33	14.8%	1.79	20	9.0%	1.41	21	9.4%	1.48
Staying at home from school or work	30	13.5%	1.47	15	6.7%	1.58	14	6.3%	1.21
Numbness, tingling in hands or feet	28	12.6%	1.50	15	6.7%	1.29	13	5.8%	1.23
Ringing in ears	24	10.8%	1.42	11	4.9%	1.29	10	4.5%	1.40

Prev, Prevalence; Int, Average Intensity

[1] Significant difference by Kruskal-Wallis test with FDR correction

[2] Significant difference by Mann-Whitney U pairwise comparison with Bonferroni correction

* p-value < 0.05; ** p-value < 0.01; *** p-value < 0.001

Appendix D. Model estimates from Bayesian adjacent category ordinal models for perceptions of work-related productivity outcomes

**Variable**	**Concentration (N = 372)**	**Relationship with Coworkers (N=372)**	**Energy (N=372)**	**Efficiency (N=372)**	**Mood at Work (N=372)**	**Interest in Work (N=372)**
	OR, [95% CrI]	*OR, [95% CrI]*	*OR, [95% CrI]*	*OR, [95% CrI]*	*OR, [95% CrI]*	*OR, [95% CrI]*
Age	OR_1 = 1.14, [0.99, 1.32] OR_2 = 0.67, [0.57, 0.79]*	OR = 0.84, [0.73, 0.98]*	OR_1 = 1.01, [0.87, 1.17] OR_2 = 0.80, [0.68, 0.93]*	OR_1 = 1.13, [0.97, 1.32] OR_2 = 0.64, [0.55, 0.75]*	OR_1 = 1.11, [0.96, 1.29] OR_2 = 0.79, [0.67, 0.94]*	OR_1 = 1.11, [0.94, 1.30] OR_2 = 0.77, [0.64, 0.91]*
BMI	OR = 1.08, [0.98, 1.19]	OR = 1.04, [0.91, 1.19]	OR = 1.07, [0.98, 1.18]	OR = 1.05, [0.95, 1.15]	OR = 1.07, [0.96, 1.18]	OR = 1.11, [0.99, 1.25]
Current contraceptive use	OR = 0.72, [0.58, 0.89]*	OR = 0.85, [0.63, 1.15]	OR = 0.93, [0.77, 1.12]	OR = 0.79, [0.64, 0.98]*	OR = 0.85, [0.68, 1.05]	OR_1 = 1.17, [0.84, 1.66] OR_2 = 0.45, [0.3, 0.66]*
Heavy bleeding experience	OR = 0.98, [0.80, 1.19]	OR = 0.91, [0.68, 1.22]	OR = 0.87, [0.72, 1.04]	OR = 0.96, [0.79, 1.17]	OR = 0.96, [0.77, 1.17]	OR = 0.90, [0.71, 1.13]
Exos employment status	OR = 1.13, [0.92, 1.37]	OR_1 = 1.83, [1.20, 2.78]* OR_2 = 0.74, [0.49, 1.11]	OR = 1.04, [0.86, 1.25]	OR_1 = 1.57, [1.16, 2.14]* OR_2 = 0.84, [0.61, 1.15]	OR = 1.03, [0.83, 1.28]	OR = 1.11, [0.88, 1.41]
Phase 2	OR = 8.50, [6.24, 11.63]*	OR = 6.96, [4.48, 10.99]*	OR_1 = 19.27, [11.67, 32.2]* OR_2 = 4.56, [2.51, 8.50]*	OR = 8.29, [6.03, 11.49]*	OR = 10.25, [7.34, 14.59]*	OR = 7.36, [5.19, 10.63]*

Phase 3	OR = 6.02, [4.49, 8.16]*	OR = 4.85, [3.14, 7.59]*	OR_1 = 22.01, [13.73, 35.25]* OR_2 = 1.92, [1.06, 3.61]*	OR = 5.08, [3.77, 6.93]*	OR = 6.70, [4.87, 9.34]*	OR = 5.44, [3.87, 7.78]*
Phase 4	OR = 1.00, [0.76, 1.31]	OR = 0.48, [0.33, 0.69]*	OR_1 = 2.10, [1.42, 3.09]* OR_2 = 0.66, [0.32, 1.35]	OR = 0.88, [0.66, 1.16]	OR = 1.16, [0.88, 1.54]	OR = 0.75, [0.56, 1.01]
Difficulty concentrating	OR = 0.82, [0.68, 0.98]*	-	-	OR = 0.96, [0.79, 1.17]	-	-
Avoid social activities	-	OR = 0.91, [0.74, 1.11]	-	-	-	-
Fatigue	-	-	OR_1 = 0.55, [0.46, 0.65]* OR_2 = 1.16, [0.97, 1.39]	-	-	-
Burst of energy or activity	-	-	OR = 1.32, [1.15, 1.52]*	-	-	-
Takes naps, stay in bed	-	-	OR = 0.87, [0.78, 0.97]*	-	-	-
Decreased efficiency	-	-	-	OR_1 = 0.50, [0.41, 0.61]* OR_2 = 1.72, [1.37, 2.16]*	-	-

Mood swings	-	-	-	-	OR = 0.77, [0.65, 0.93]*	-
Staying at home from school or work	-	-	-	-	-	OR = 0.78, [0.67, 0.91]*
MDQ concentration subscale	OR_1 = 0.48, [0.38, 0.61]* OR_2 = 1.97, [1.57, 2.50]*	-	-	OR = 0.87, [0.69, 1.08]	-	OR_1 = 0.71, [0.56, 0.88]* OR_2 = 1.30, [0.97, 1.73]
MDQ behavior change subscale	-	OR_1 = 0.63, [0.47, 0.84]* OR_2 = 1.05, [0.72, 1.52]	-	-	-	OR_1 = 0.53, [0.41, 0.69]* OR_2 = 1.31, [0.94, 1.79]
MDQ negative subscale	-	OR_1 = 0.63, [0.49, 0.82]* OR_2 = 1.14, [0.81, 1.59]	-	-	OR_1 = 0.52, [0.42, 0.65]* OR_2 = 1.38, [1.08, 1.76]*	-
MDQ arousal subscale	-	OR_1 = 1.06, [0.84, 1.33] OR_2 = 1.58, [1.33, 1.88]*	OR_1 = 0.68, [0.56, 0.83]* OR_2 = 1.36, [1.13, 1.63]*	-	OR_1 = 0.90, [0.77, 1.07] OR_2 = 1.58, [1.36, 1.84]*	-

OR, Odds Ratio; OR_1, Odds Ratio comparing negative vs. neutral impact in the adjacent category ordinal model; OR_2, Odds Ratio comparing positive vs. neutral impact in the adjacent category ordinal model; CrI: Credible Interval

* 95% credible intervals that exclude 1 (suggesting a consistent non-null effect)

- Not included in model
